# Phylogeny of Immune Recognition: Processing and Presentation of Structurally Defined Proteins in Channel Catfish Immune Responses

**DOI:** 10.1155/1991/32534

**Published:** 1991

**Authors:** Abbe N. Vallejo, Norman W. Miller, L. William Clem

**Affiliations:** Department of Microbiology University of Mississippi Medical Center 2500 N. State Street Jackson Mississippi 39216-4505 USA

## Abstract

This work was undertaken to investigate whether or not antigen processing and presentation are important in channel catfish in vitro secondary immune responses elicited with structurally defined proteins, namely, pigeon heart cytochrome C (pCytC), hen egg lysozyme, and horse myoglobin. The use of *in vitro* antigen-pulsed and fixed B cells or monocytes as antigen presenting cells (APC) resulted in autologous peripheral blood leukocytes (PBL) responding with vigorous proliferation and antibody production *in vitro*. In addition, several long-term catfish monocyte lines have been found to function as efficient APC with autologous but not allogeneic responders. Subsequent separation of the responding PBL into sIg^-^ (T-cell-enriched) and B (sIg^+^) cell subsets showed that both underwent proliferative responses to antigen-pulsed and fixed APC. Moreover, allogeneic cells used as APC were found to induce only strong mixed leukocyte reactions without specific *in vitro* antibody production. Initial attempts at identifying the immunogenic region(s) of the protein antigens for catfish indicated there are two such regions for pCytC, namely, peptides 66-80 and 81-104.


*Developmental Immunology*, 1991, Vol. 1, pp. 137–148
Reprints available directly from the publisher
Photocopying permitted by license only

© 1991 Harwood Academic Publishers GmbH
Printed in the United Kingdom

### Phylogeny of Immune Recognition: Processing and Presentation of Structurally Defined Proteins in Channel Catfish Immune Responses

ABBE N. VALLEJO, NORMAN W. MILLER, and L. WILLIAM CLEM*

*Department of Microbiology, University of Mississippi Medical Center, 2500 N. State Street, Jackson, Mississippi 39216-4505*

This work was undertaken to investigate whether or not antigen processing and presentation are important in channel catfish *in vitro* secondary immune responses elicited with structurally defined proteins, namely, pigeon heart cytochrome C (pCytC), hen egg lysozyme, and horse myoglobin. The use of *in vitro* antigen-pulsed and fixed B cells or monocytes as antigen presenting cells (APC) resulted in autologous peripheral blood leukocytes (PBL) responding with vigorous proliferation and antibody production *in vitro*. In addition, several long-term catfish monocyte lines have been found to function as efficient APC with autologous but not allogeneic responders. Subsequent separation of the responding PBL into $sIg^-$ (T-cell-enriched) and B ($sIg^+$) cell subsets showed that both underwent proliferative responses to antigen-pulsed and fixed APC. Moreover, allogeneic cells used as APC were found to induce only strong mixed leukocyte reactions without specific *in vitro* antibody production. Initial attempts at identifying the immunogenic region(s) of the protein antigens for catfish indicated there are two such regions for pCytC, namely, peptides 66-80 and 81-104.

KEYWORDS: APC, antigen presentation, antigen processing, catfish.

### INTRODUCTION

In recent years, the use of globular proteins has facilitated the understanding of molecular aspects of immune recognition and genetic control of immune responses in mammals (Sercarz and Berzofksy, 1987; Smith-Gill and Sercarz, 1989). Proteins with known amino-acid sequences and spatial structures, such as lysozyme, myoglobin, and cytochrome C, have become effective tools in determining the fine specificities of both B- and T-cell repertoires. Furthermore, the availability of species variants of these proteins has permitted the exact identification of antigenic determinants by comparing the cross reactivities of either the native forms of the proteins or their peptide fragments derived by chemical or enzymatic cleavage as well as chemical synthesis. This latter experimental approach is becoming useful for identifying the critical contact points of the peptide antigen with the major histocompatibility complex (MHC) molecule and the T-cell receptor (Heber-Katz et al., 1983). Along these lines, we have conducted studies to further elucidate the mechanisms of immunity of a phylogenetically lower ectothermic vertebrate, the channel catfish (*Ictalurus punctatus*). We have previously reported (Vallejo et al., 1990b) that the generation of secondary *in vitro* immune responses to complex thymus-dependent antigens in channel catfish requires steps akin to antigen processing and presentation in mammals (Pernis et al., 1988; Schook and Tew, 1988). The present work now extends these observations to *in vitro* immune responses to well-characterized protein antigens. Furthermore, the development of long-term catfish monocyte lines (Vallejo et al., 1990a) has facilitated studies on the identification of the responding lymphocyte populations, putative immune restriction, and presentation of peptide fragments by fixed antigen-presenting cells (APC).

*Corresponding author.

### RESULTS

### Channel Catfish Exhibit Secondary *In Vitro* Immune Responses to Structurally Defined Proteins

We have previously reported that catfish can immunologically discriminate between two species variants of a complex thymus-dependent antigen, hemocyanin (Vallejo et al., 1990b). In order to determine whether or not the same specificity occurs with simpler well-characterized proteins, groups of five fish were immunized with pigeon heart cytochrome C (pCytC), hen egg lysozyme (HEL) and horse muscle myoglobin (EqMb). When peripheral blood leukocytes (PBL) from these fish were cultured in the presence of soluble antigen, vigorous antigen-specific *in vitro* proliferation was observed only with immune PBL stimulated with homologous but not heterologous antigen (as depicted for a representative fish from each group in Fig. 1). Similarly, specific antibodies produced *in vitro* were detected only in supernatants of cultures stimulated with homologous antigen. There were no detectable antibodies from unstimulated controls or cultures stimulated with heterologous antigen.

### Catfish APC Function Can Be Accomplished by Monocytes and T and B Cells

As previously reported (Vallejo et al., 1990b), antigen processing and presentation by a variety of cells were important in the generation of secondary *in vitro* proliferative and antibody responses to hemocyanins. Such observations raised the question as to whether or not similar events were involved in responses to simpler protein antigens. By using two different EqMb-primed fish as representative animals, results showed the EqMb-pulsed monocytes and B cells (i.e., surface/membrane immunoglobulin-positive [$sIg^+$] cells) each induced *in vitro* proliferation of autologous PBL comparable to cultures stimulated with soluble EqMb plus unpulsed APC (Fig. 2). Moreover, positively selected B cells or negatively selected B cells treated with anticatfish Ig (monoclonal antibody [mAb] 9E1) prior to antigen pulsing resulted in the vigorous proliferation of responding PBL. The magnitudes of such responses were almost twice those generated with antigen-presenting B cells that were negatively selected without incubation with anticatfish Ig. On the other hand, positively or negatively selected $sIg^-$ cells (i.e., T-cell-enriched) incubated with anticatfish pan T/thrombocyte (mAb 13C10) were not effective APC in eliciting proliferative responses. However, it should be noted that cultures stimulated with antigen-pulsed $sIg^-$ cells (T-cell-enriched) showed $^3$H-thymidine uptake significantly higher than either the unstimulated controls or PBL cocultured with unpulsed/fixed APC. As in the previous experiments, APC fixed prior to antigen pulsing did not stimulate autologous responders.

### Long-Term Catfish Monocyte Lines Are Effective APC

We have previously described the development of long-term monocyte lines from channel catfish (Vallejo et al., 1990a). In the present work, such cell lines were used as APC. Since the fish serving as sources of these cell lines were still alive, it was possible to determine the APC function of these lines with autologous responder PBL. Results indicated that cell lines used as APC that had been pulsed with homologous antigen and subsequently paraformaldehyde-fixed induced proliferation and antibody production of autologous PBL *in vitro* (Fig. 3). However, the use of autologous cell lines pulsed with heterologous antigen stimulated neither proliferation nor antibody production by autologous responders (data not shown). Similarly, as in previous experiments, prefixed and antigen-pulsed autologous cell-line APC were nonstimulatory.

### Catfish Immune Responses Appear To Be Restricted

In order to ascertain whether or not allogeneic cells could be used as APC, experiments involving 17 (out of a possible 72) pairwise allogeneic combinations of 6 catfish monocyte lines (as APC) and PBL (as responders) from 12 immunized (3 HEL-, 4 EqMb-, and 5 pCytC-immune) fish were conducted. In each case (as depicted in Fig. 4 for a representative fish), the fixed allogeneic cell line elicited vigorous *in vitro* proliferative responses, presumably akin to mixed leukocyte reactions (MLR). These responses were, however, not further increased by antigen pulsing of the APC. Furthermore, no detectable specific antibodies were found in such allogeneic situations unless soluble homologous antigen was added directly to the culture, that is, presumably as a consequence of the presence of autologous APC in the responding PBL.

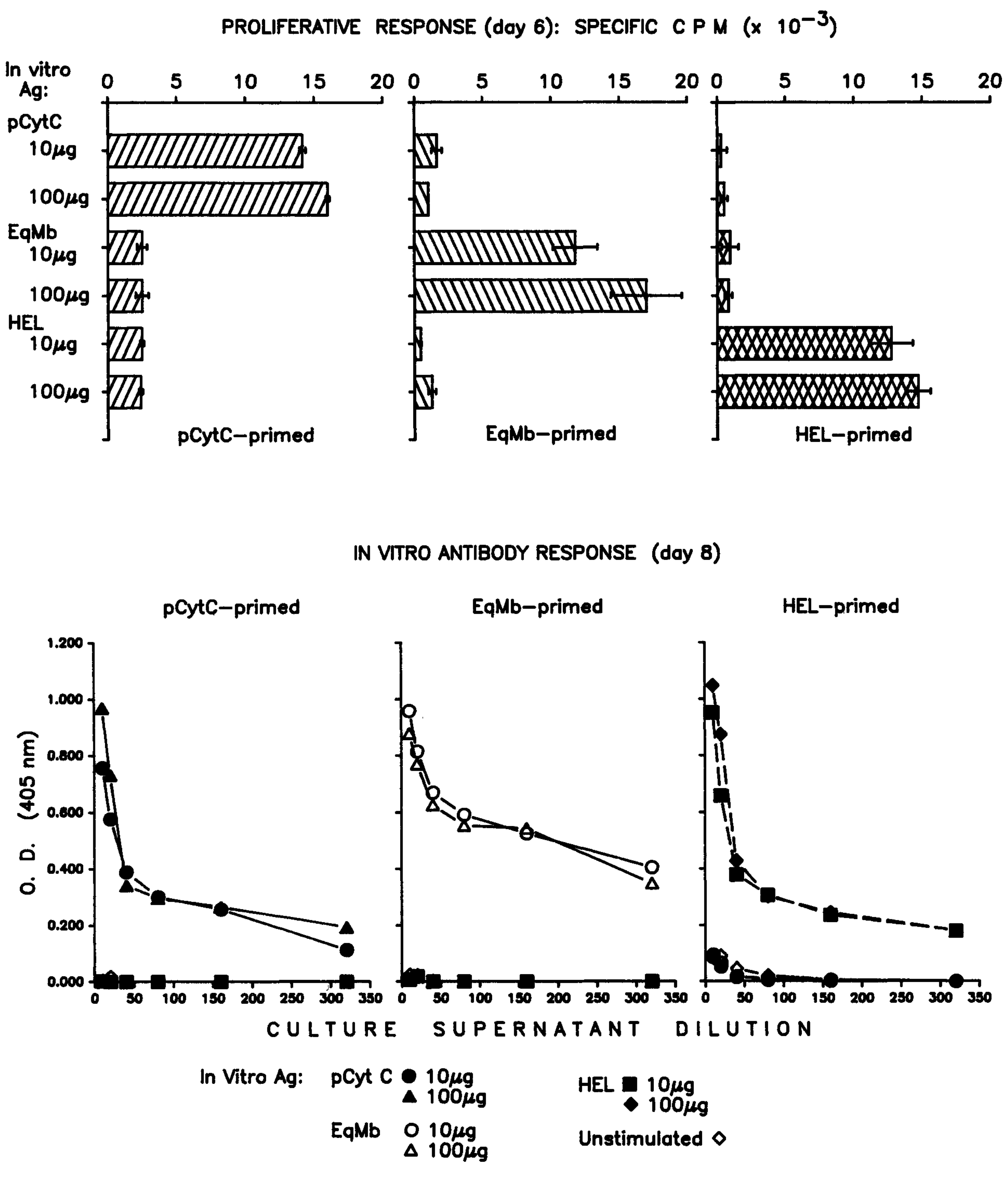

FIGURE 1. Proliferative and antibody responses of fresh unfractionated PBL from fish primed *in vivo* with antigen and stimulated *in vitro* with various concentrations of soluble homologous or heterologous antigen. Background cpm of unstimulated cultures in each experiment were ⩽4000. Values indicated were means of triplicate cultures. S.D. in each case was ⩽15% of the mean.

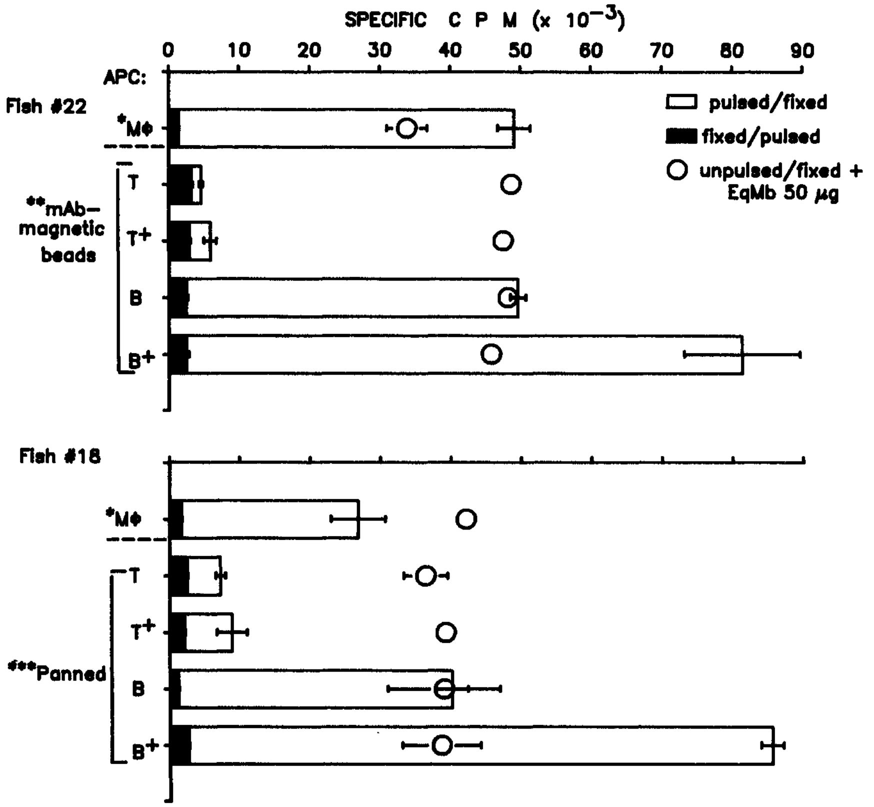

*Fibronectin-adherent, NSE+ cells

**Negatively selected lymphocytes by mAb-magnetic beads

T – B cell (9E1)-depleted

T+ – B cell depleted + 1 hr. incubation with 13C10

B – T cell (13C10)-depleted

B+ – T cell depleted + 1 hr. incubation with 9E1

***Lymphocytes separated by panning with mAb

T – 9E1 Non-adherent

T+ – 13C10 Adherent

B – 13C10 Non-adherent

B+ – 9E1 Adherent mAbs: 9E1 – anti-catfish Ig; 13C10 – anti-catfish pan T

FIGURE 2. Proliferative responses of PBL from two EqMb-primed fish (numbers 22 and 18) co-cultured with autologous monocytes (Mø), sIg⁻ (T-cell-enriched) or B (sIg⁺) cells as APC. Background cpm of unstimulated cultures or cultures with unpulsed/fixed APC was ≤5000.

### Both Catfish T and B Cells Undergo Proliferation *In Vitro*

The APC function of long-term catfish monocyte lines was explored further to ascertain which responding lymphocyte population was induced to proliferate *in vitro*. Results showed both sIg⁻ (T-cell-enriched) and B (sIg⁺) cells underwent proliferation in response to autologous antigen-pulsed cell-line APC (Fig. 5). Further, such *in vitro* responses were blocked by compounds such as chloroquine and leupeptin. However, sIg⁻ cells did not proliferate as vigorously as B cells in response to soluble antigen (in the absence of APC) although these sIg⁻ cell *in vitro* proliferative responses were significantly higher than those of unstimulated controls or sIg⁻ cells cocultured with unpulsed and fixed APC.

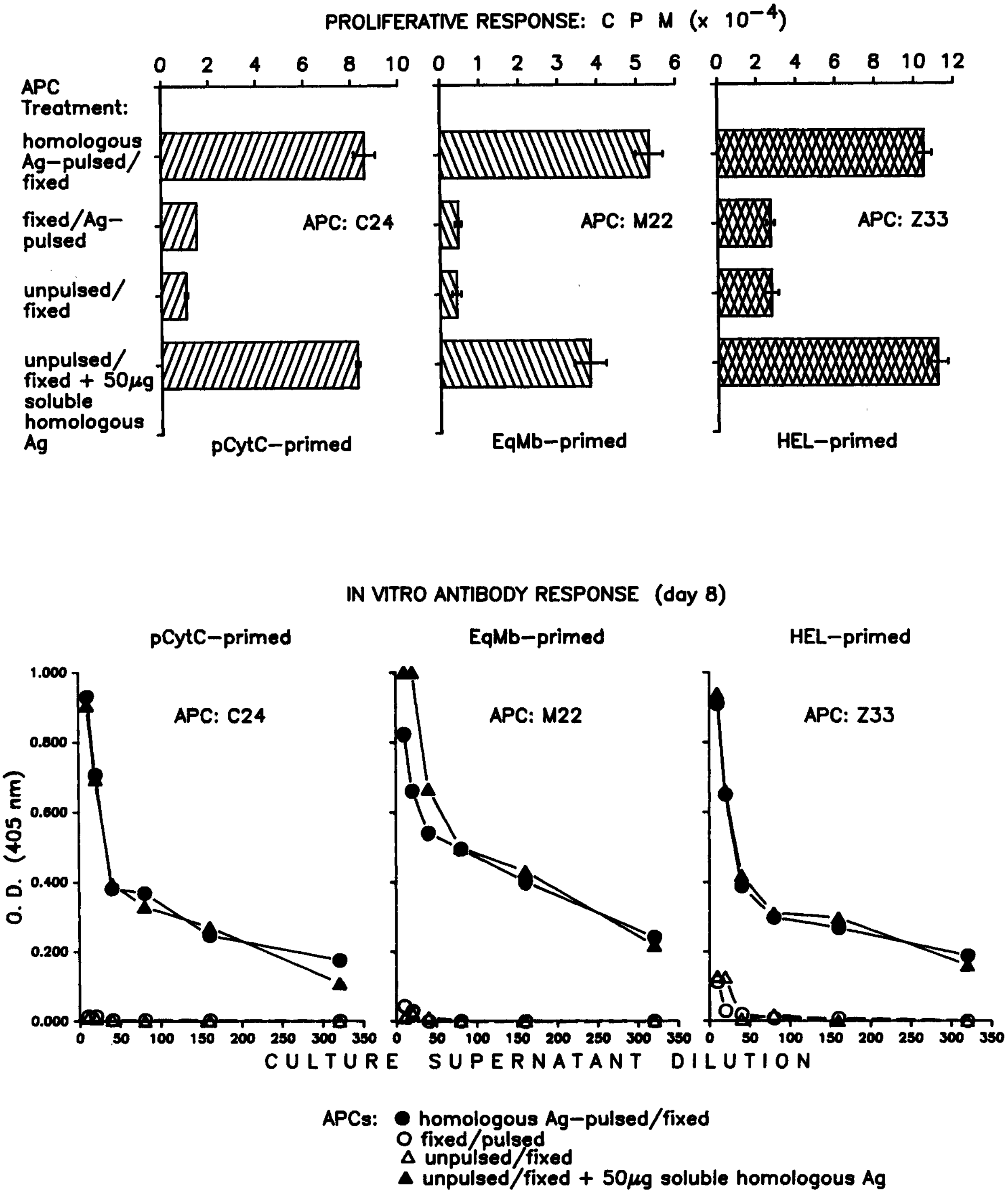

FIGURE 3. Proliferative and antibody responses of fresh PBL from antigen-primed fish co-cultured with variously treated autologous long-term catfish monocyte lines as APC. Values indicated were means of triplicate cultures. S.D. in each case was ≤12% of the mean.

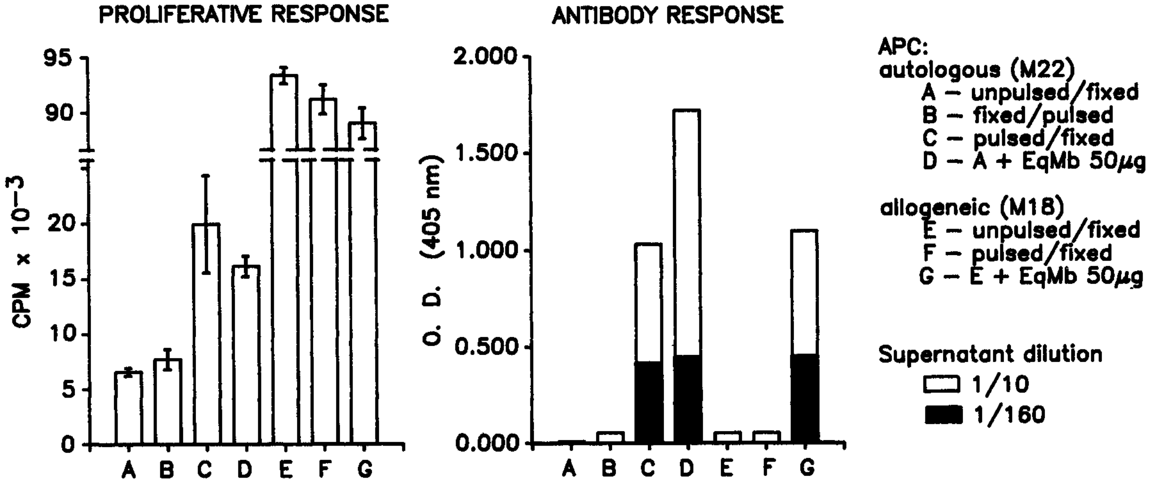

FIGURE 4. Proliferative and antibody responses of PBL from EqMb-primed fish co-cultured with variously treated autologous or allogeneic long-term monocyte lines as APC. Values indicated were means of triplicate cultures. S.D. in each case was ≤18% of the mean.

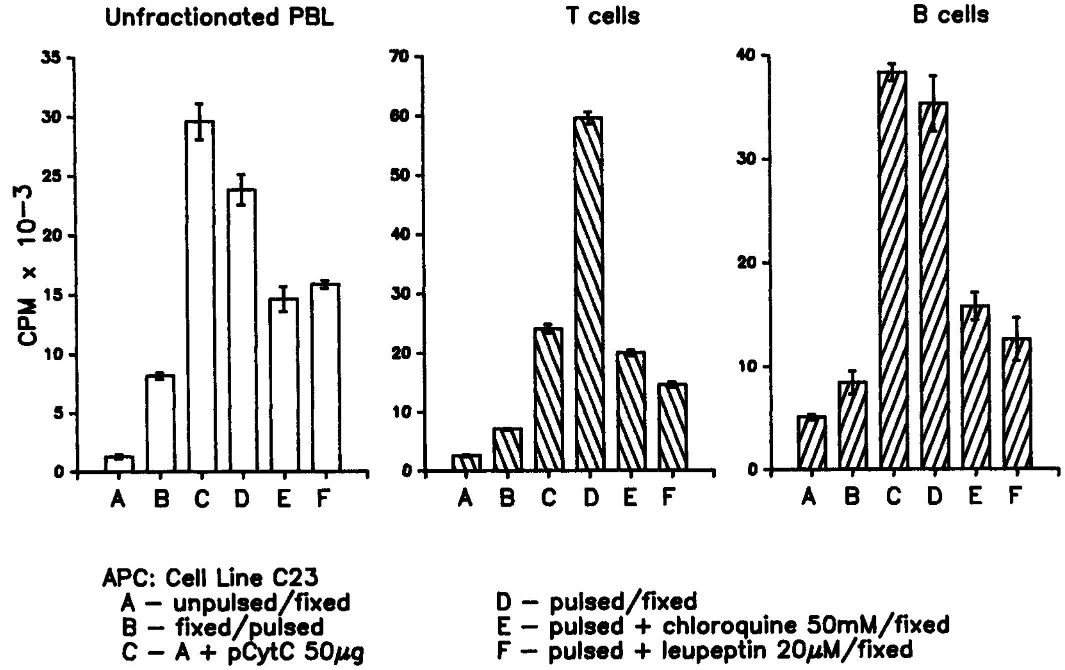

FIGURE 5. Proliferative responses of PBL, negatively selected sIg$^-$ (T-cell-enriched) and B (sIg$^+$) cells from pCytC-primed fish co-cultured with autologous monocyte line as APC. Antigen pulsing of APC was carried out with or without inhibitors at the indicated nontoxic concentration. Values indicated were means (±S.D.) of triplicate cultures.

### The Ability of Antigen-Pulsed APC to Stimulate Immune PBL is Correlated with Antigen Uptake During Pulsing

The ability of antigen-pulsed and fixed unfractionated PBL, blood monocytes, B cells, and long-term fish cell lines to stimulate autologous responders *in vitro* raised the question as to how much protein antigen became cell-associated during the pulsing period. By using fish monocyte lines as model APC, cells were incubated with $^{14}CH_3$-protein. It should be noted that this modification of protein antigens did not affect the specificity of the *in vitro* proliferative responses of immune PBL (data not shown). Results of antigen-uptake assays showed that the monocyte lines incorporated about 12 to 23% of the total protein added during a 7-h pulsing period with or without inhibitors. There was an average uptake of 95 $\mu$g protein/$10^8$ cells for $^{14}CH_3$-pCytC and $^{14}CH_3$-EqMb, and 72.5 $\mu$g protein/$10^8$ cells for $^{14}CH_3$-HEL. Furthermore, variable (10-20%) amounts of cell-associated proteins were found to be degraded to smaller peptide fragments as assessed by SDS-PAGE and autoradiography (data not shown). On the other hand, prefixed cells exhibited negligible uptake of radiolabeled protein ($\leq$1.5% or $\leq$7.5 $\mu$g protein/$10^8$ cells).

### Requirement of Live APC During Antigen Pulsing Is Bypassed with the Presentation of "Preprocessed" Peptides by Prefixed APC

The previous experiments showed that APC prefixed prior to antigen pulsing were not stimulatory to immune PBL. A study was therefore conducted to determine whether or not fragments of a structurally defined protein, pCytC, could be presented by prefixed APC. Results showed that such was the case (Fig. 6, for the representative three fish studied in this regard). Incubation of prefixed cell-line APC with pCytC peptide fragments corresponding to residues 1-80, 66-104, 66-80, and 81-104 were found to be stimulatory to autologous PBL as observed when native pCytC-pulsed APC were used. However, the magnitude of the responses due to peptides 66-104, 66-80, and 81-104 were significantly higher than for peptide 1-80 or the native protein. On the other hand, peptide 1-65 was clearly nonstimulatory.

### DISCUSSION

This work embodies findings that elucidate our knowledge of antigen recognition relevant to teleost immunity. Firstly, results reported herein show the exquisite specificity of the secondary *in vitro* immune responses to channel catfish to well-characterized proteins such as pCytC, HEL, and EqMb. The use of such protein antigens in studies utilizing mammalian models allowed the determination of the fine specificities of the immunologic repertoire. Such studies have revealed that the levels of responsiveness to a particular antigen (i.e., high, low, or nonresponsiveness) are not simply due to holes in the repertoire, suppressor activities, or anergy (Cease et al., 1986; Berzofsky, 1988), but are largely influenced by factors intrinsic to the antigen itself and by cellular processing of the antigen. Apparently, the observed skewing of T- and B-cell responses is due to the limited number of epitopes that can be presented by MHC molecules (Kojima et al., 1988) and, hence, the level of responsiveness varies among the epitopes used. In other words, one epitope (the "immunodominant" one, hence the concept of epitope immunodominance) may elicit a greater magnitude of response compared to the rest of the epitopes (Shastri et al., 1985; Berzofsky, 1988). Although the applicability of this concept to teleost immunity remains to be demonstrated, the finding that pCytC-immune catfish PBL can proliferate *in vitro* in response to prefixed, pCytC peptide-pulsed autologous APC suggests that the same phenomenon might be occurring in fish (discussed further later). Significantly, the results also show that the polyclonal proliferative responses due to either peptides 66-80 or 81-104 are greater than the responses to peptide 1-80. Whether or not the same catfish lymphocyte clonotypes are responding to each of the peptides remains to be investigated.

Secondly, this work demonstrates that the generation of catfish secondary *in vitro* immune responses involves steps akin to antigen processing and presentation in mammals, and that both monocytes and B cells are effective APC similar to the situation in mammals (Gosselin et al., 1988; Pierce et al., 1988; Schook and Tew, 1988; Vallejo et al., 1990a, 1990b). The use of antigen-pulsed and paraformaldehyde-fixed APC elicited *in vitro* proliferation and antibody production by autologous immune PBL. Clearly, such responses must be attributed to antigen that became cell-associated during pulsing and was presumably processed intracellularly. Furthermore, subsequent fixation of APC must have retained the antigen in a form that is recognizable by responding lymphocytes. In addition, the APC function of fish B cells is enhanced when they are isolated by direct panning using anticatfish Ig (mAb 9E1) or by negative selection with subsequent incubation with anticatfish Ig. Studies indicate that such treatments of B cells, both in mammals and catfish, result in their activation (Cambier et al., 1987; Miller et al., in preparation). On the other hand, sIg$^-$ cells (T-cell-enriched), either positively or negatively selected and incubated with anticatfish pan T/thrombocyte (mAb 13C10), are not efficient APC. Whether or not such treatment of catfish sIg$^-$ cells results in the activation of these cells is not clear since the cell determinant recognized by the mAb (13C10) is not yet functionally defined (Miller et al., 1987). However, it should be noted that the *in vitro* proliferative responses of immune PBL to autologous sIg$^-$ cells used as APC are significantly higher than the unstimulated controls or PBL co-cultured with unpulsed/fixed APC. It is most unlikely that such observations are due to contaminating B cells among the APC considering the high level of purity of the sIg$^-$ (T-cell-enriched) cell preparations (although it cannot be ruled out that sIg$^-$ cells other than T cells may be involved). Nonetheless, the original hypothesis that fish T cells also present antigen albeit less efficiently than either B cells or monocytes (Vallejo et al., 1990b), is more plausible and perhaps more appealing. Although MHC (or MHC-like) molecules have yet to be described for fish, previous studies showed that fish T cells can elicit weak MLR (Miller et al., 1986). If this MLR is attributable in part to MHC, and if MHC is involved in antigen presentation in catfish immune responses, ascribing APC function to fish T cells does not conflict with the immunologic paradigm of MHC-"processed" antigen interaction as a requisite for T-cell recognition (Berzofsky, 1988).

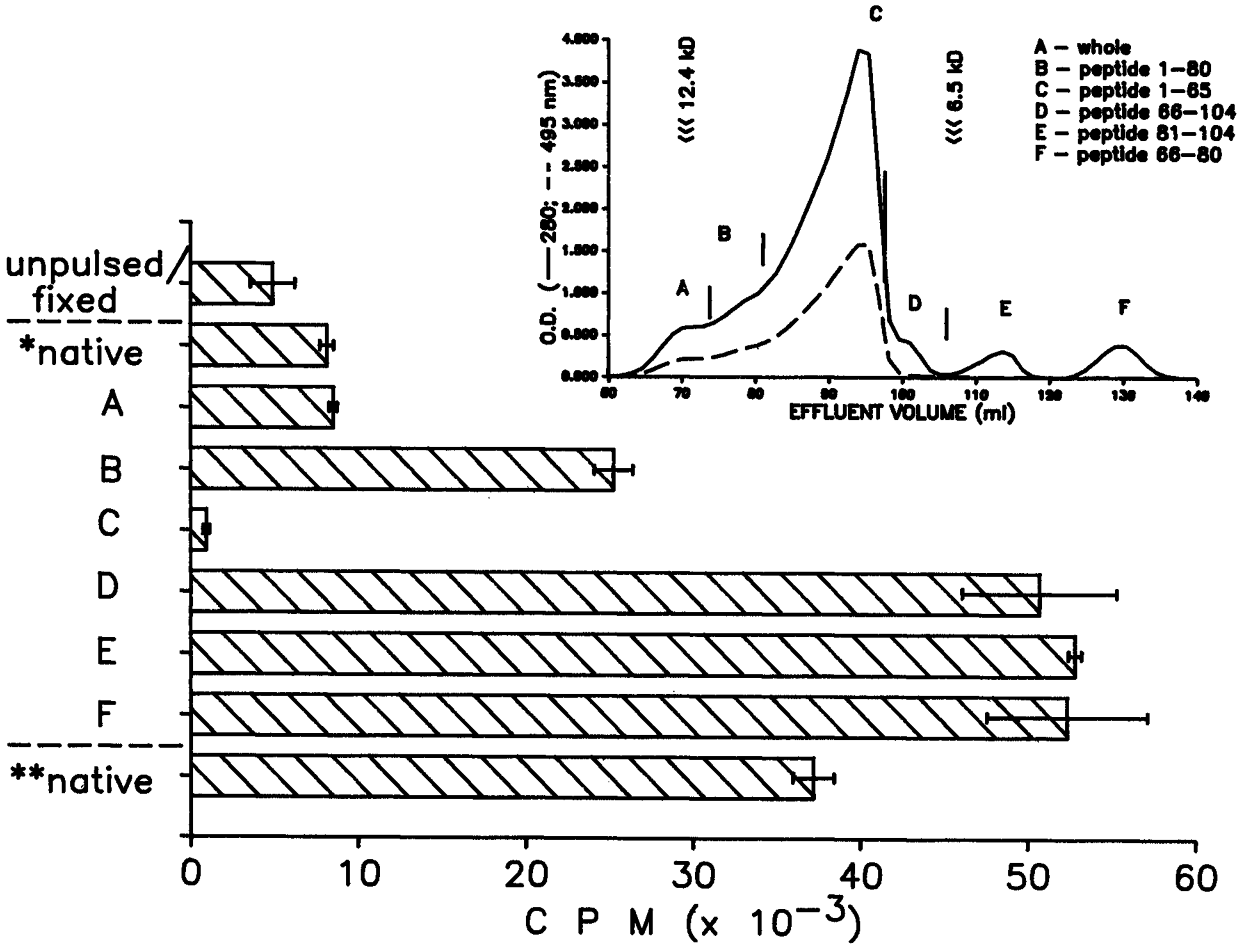

FIGURE 6. Proliferative responses of monocyte-depleted PBL from pCytC-primed fish co-cultured with autologous cell line as APC. Prefixed (*) monocyte line C4 cells were incubated with native or unfragmented intact (A) pCytC, peptide fragments (B–F) or live C4 cells (**) pulsed with native pCytC. Values indicated were means ( ± S.D.) of triplicate cultures. (*Top inset*) A typical Sephadex G50 elution curve of CNBr-fragmented pCytC.

This work also demonstrates the utility of long-term fish cell lines in studying accessory cell functions. A previous study (Vallejo et al., 1990a) indicated that these cell lines, which are morphologically similar to mammalian monocytes, have APC function in addition to the ability to produce interleukin-1. In all assays conducted, antigen-pulsed and fixed long-term fish monocytes used as APC have consistently induced specific *in vitro* proliferative and antibody responses by autologous PBL as responders. Further, addition of compounds such as chloroquine and leupeptin during antigen pulsing resulted in the reduction of the *in vitro* responses. These compounds are known to block antigen processing as well as interfere with Class II MHC assembly by mammalian accessory cells and it seems likely that they interfere with antigen processing by fish APC as well. Moreover, results show that both catfish $sIg^-$ (T-cell-enriched) and B ($sIg^+$) cells underwent proliferation in response to autologous cell-line APC. This latter observation indicates that in mixed cultures of PBL and monocyte-line APC, antibody production *in vitro* must be due to T helper cells effecting B-cell differentiation as well as T and B-cell proliferation. Moreover, data obtained also show that fish B cells can respond to soluble antigen similar to the situation in mammals. Cross linking of Ig receptors on B cells by antigen can result in the activation and proliferation of such antigen-specific B cells (Cambier et al., 1987). Further, since B cells also have accessory cell functions (Pierce et al., 1988), soluble antigen is presumably being processed and subsequently interacts with idiotypic B cells, which then become activated and, if T-cell-derived B-cell growth factors are present, can undergo differentiation. On the other hand, fish $sIg^-$ cells (T-cell-enriched) show low proliferative responses to soluble antigen, albeit significantly higher than the unstimulated controls. Consistent with the previously mentioned notion that fish T cells may have the ability to process and present antigen, such observations may not be particularly surprising. T cells in culture may be processing antigen and present fragments thereof to idiotypic T cells. In mammals, it is generally accepted that unlike B cells, T lymphocytes do not usually directly interact with native protein antigens but rather with the processed antigen in the context of "self" MHC (Buus et al., 1987). However, there are several reports that unequivocally demonstrated the presence of MHC Class II molecules on T cells of various vertebrates ranging from humans to birds to amphibians (Fu et al., 1978; Crepaldi et al., 1986; Flajnik et al., 1990; Kroemer et al., 1990) and thus the possibility that these T cells could have APC function cannot be ignored.

Studies employing long-term catfish monocyte lines as APC further demonstrated that immune restriction likely occurs in fish. Allogeneic mixtures of cell-line APC and immune PBL yield only strong MLR but not specific antibody responses *in vitro* unless soluble antigen is added to the cultures. Such observations suggest two other important aspects of teleost immunity, namely, (a) fish alloantigens are unequivocally present, although their complexity and relationship to known vertebrate MHC molecules remains to be elucidated; and (b) the generation of secondary *in vitro* antibody responses depends both on the antigen and the APC used. As alluded to earlier, alloantigens (at least in mammals) are responsible for MLR, and this *in vitro* event is attributable to MHC molecules (Lechler et al., 1990). The precise mechanism wherein immune catfish PBL are not induced to produce specific antibodies *in vitro* despite vigorous MLR due to allogeneic antigen-pulsed APC is not clear at this time. However, by analogy with mammalian systems, this phenomenon (i.e., vigorous MLR but no specific antibody production *in vitro*) may be attributable to at least one of several possibilities. Assuming that MHC-like molecules are involved in antigen presentation in catfish immune responses, it is possible that such mixed cultures of cell-line APC and immune PBL responders are completely allogeneic (i.e., absence of shared MHC-like determinants). In this situation, allo-MHC might not bind the processed antigen in precisely the same manner as "self" MHC (i.e., constraint of MHC restriction) and thus T-inducer/effector cells would not be activated and able to help B cells. It is also possible that such cultures are "semiallogeneic" (i.e., they have at least one shared MHC-like determinant). If this is the case, the lack of specific antibody responses *in vitro* may be due to some form of inhibition that can only be speculated on at this time.

Finally, the present work suggests that the generation of fish immune responses requires antigen processing events similar to the situation in mammals (Pernis et al., 1988; Schook and Tew, 1988). Fish APC appear to take up and degrade antigen and in so doing may expose epitopes that might be normally buried in the native conformation of the protein antigen. This notion is further supported by experiments involving the presentation of protein fragments by fixed APC, thereby circumventing the requirements for intracellular processing. Data obtained show that four pCytC peptides are as immunogenic as "naturally" processed pCytC to immune PBL. Further, the magnitude of responses to peptides 66-80 and 81-104 are similar to responses to peptide 66-104, but considerably greater than the responses to peptide 1-80. Peptide 1-65 however, appears inhibitory. These observations warrant three comments: (a) processing of pCytC is important in the generation of specific *in vitro* immune responses in channel catfish, (b) there are at least three immunogenic regions in pCytC recognized by catfish PBL, and (c) catfish polyclonal response to pCytC is seemingly focused to an immunodominant epitope lying within peptide 66-104. As previously discussed, epitope immunodominance can govern the degree of responsiveness to a given protein antigen in mammalian systems (Shastri et al., 1985; Berzofsky, 1988; Kojima et al., 1988) and it is most likely that the same is true for catfish. The finding that peptide 66-104 is immunogenic suggests that there might be a cluster of epitopes within this region and that peptides 66-80 and 81-104 represent two of them in addition to peptide 1-80. Parenthetically, in mice, it is peptide 81-104 that is considered immunodominant (Collawn et al., 1989). The inhibitory effect of peptide 1-65 for catfish responses is unexplained and whether or not heme, a large proportion of which co-elutes with this peptide, has a role can only be speculated. Clearly, studies are needed to determine the clonotypic specificities of catfish T and B cells with respect to pCytC as well as the minimal stimulatory ("core") epitope within peptide 66-80 and/or 81-104.

### MATERIALS AND METHODS

### Experimental Animals

The acquisition, laboratory maintenance, and immunization of channel catfish have been previously described (Faulman et al., 1983; Miller and Clem, 1984; Vallejo et al., 1990b).

### Antigens and Inhibitors pCytC, HEL, EqMb, chloroquine, and leupeptin were obtained from Sigma (St. Louis, MO). Stock solutions were prepared at 10 mg protein/ml in phosphate-buffered saline or at 1.0 M for chloroquine and leupeptin and stored at −20°C.

Peptide fragments of pCytC were also used as antigens. Peptides were derived by cyanogen bromide cleavage of pCytC according to the method of Corradin and Harbury (1970), purified by two cycles of gel filtration on a Sephadex G50 column (1.5 ×150 cm; Pharmacia Fine Chem. Inc., Piscataway, NJ), dialyzed against distilled water, and concentrated by rotary evaporation.

### Antigen-Presenting Cells

Freshly isolated monocytes, $sIg^-$ (T-cell-enriched) and B ($sIg^+$) cells were used as APC. PBL were isolated from whole blood by centrifugation over Ficoll-Hypaque (Lymphoprep, Accurate Chemical Science Corp., Westbury, NY) as previously described (Miller and Clem, 1988). Isolation of monocytes by adherence to fibronectin-coated plates and panning protocols for the positive selection of catfish T and B cells were also carried out as previously described (Sizemore et al., 1984; Vallejo et al., 1990b). In addition, negatively selected T and B cells were obtained by reciprocal depletion of $sIg^+$ and $sIg^-$ cells, respectively, using magnetic beads. Monocyte-depleted PBL (see what follows) were incubated with either mAb 9E1 (anticatfish Ig) or 13C10 (antipan T/thrombocyte) (Miller et al., 1987) for 1 h at 4°C, centrifuged, and washed. The cell pellet was incubated with goat antimouse Ig conjugated to magnetic beads (Dynabeads M-450, Dynal Inc., Great Neck, NY) for an additional hour at 4°C. The efficacy of this negative-selection protocol is illustrated by the finding that depletion of 13C10-reactive cells from PBL yielded B ($sIg^+$-cell preparations (i.e., unmagnetized cells) with >97% purity as revealed by routine immunofluorescence assay by flow cytometry. Moreover, mitogen assays showed that these cells, with or without additional monocytes, responded to lipopolysaccharide (LPS) but not to concanavalin A (Con A) as expected (Sizemore et al., 1984). Similarly, depletion of 9E1-reactive cells from PBL also yielded negatively selected $sIg^-$ cells with >97% purity (as assessed with mAb 13C10). Although these latter cell preparations were not exclusively T cells (i.e., ≈10% thrombocytes and ≤2% granulocytes and plasma cells), they are clearly T-cell-enriched. As expected, mitogen assays with these cells did not show proliferative responses to Con A in the absence of monocytes, nor did they respond to LPS with or without additional monocytes. These negatively selected cells were then immediately pulsed with antigen or incubated with mAbs 9E1 or 13C10 for 1 h at 27°C prior to pulsing with antigen. Long-term catfish monocyte lines were also used as APC. These cell lines have been previously characterized and their APC function ascertained (Vallejo et al., 1990a).

### Antigen Processing/Presentation Assay

Protocols for antigen pulsing of APC were described previously (Vallejo et al., 1990b). In this study, however, APC were incubated with the native antigen (i.e., 500 $\mu g/10^7$ cells/ml) for 5 to 8 h at 27°C, the optimum time based on initial assays. Cells were subsequently washed and fixed with 0.5% paraformaldehyde for 15–30 min at room temperature. Antigen-pulsed/fixed APC were extensively washed, allowed to incubate in fresh medium for 1 h at room temperature, and then given a final wash before coculturing with responder cells. Antigen pulsing in the presence of inhibitors at the indicated maximum nontoxic concentration was also carried out as described previously.

In assays involving the pCytC peptides, long-term monocyte lines were used as APC. Approximately 100 $\mu g$ of the peptide were added to $10^7$ paraformaldehyde-fixed cells and incubated at 4°C with gentle agitation for 18 to 24 h. The cells were washed and cultured with autologous responders.

Responders in all assays were either whole PBL or monocyte-depleted PBL, except where negatively selected $sIg^-$ (T-cell-enriched) or B ($sIg^+$) cells were used as indicated. Depletion of blood monocytes was carried out by incubating PBL in a P-6 column (EconoPac 10DG, BioRad, Richmond, CA) for 1 h at 27°C and then eluting the nonadherent cells (Vallejo et al., 1990b). Negative selection of T and B cells using mAb-magnetic beads was as described before. Triplicate cultures of $10^6$ cells were stimulated with soluble antigen at the indicated concentrations or with antigen-pulsed/fixed autologous or allogeneic APC (approximately $5 \times 10^5$). The culture system for catfish cells has been described elsewhere (Miller and Clem, 1988). Assays for $^3H$-thymidine uptake after 6 days and *in vitro* antibody response by enzyme-linked immunoabsorbent assay (ELISA) after day 8 in culture were carried out as previously described (Vallejo et al., 1990b).

### Antigen Uptake pCytC, HEL, and EqMb were uniformly labeled by reductive methylation with $^{14}C$-formaldehyde and sodium cyanoborohydride (Sigma) (Dottavio-Martin and Ravel, 1978). $^{14}CH_3$-protein with or without inhibitors was added to catfish monocyte lines and incubated at 27°C. At various times, the cells were washed extensively with complete medium and lysed with 100 $\mu l$ of a hypotonic buffer containing 50 mM Tris-HCl, pH 7.0; 10 mM NaCl; 1% NP40; 1 mg/ml aprotinin; 1 mM PMSF; 5 mM EDTA. The cell-associated radioactivity was determined by liquid scintillation spectrometry using a nontoluene fluor (EcoScint A, National Diagnostics, Manville, NJ) and degradation was assessed by SDS-urea-PAGE (Burr and Burr, 1983).

### ACKNOWLEDGMENTS

This work was supported in part by a grant from the U.S. National Institutes of Health (#5-R37-AI-19530).

*(Received September 12, 1990)*

*(Accepted September 17, 1990)*

### REFERENCES

Berzofsky J.A. (1988) Structural basis of antigen recognition by T lymphocytes: Implications for vaccines. J. Clin. Invest. **82:** 1811-1817.

Burr F.A., and Burr, B. (1983) Slab gel system for the resolution of oligopeptides below molecular weight of 10,000. Methods Enzymol. **96:** 239–244.

Buus S., Sette A., Colon S.M., Miles C., and Grey H.M. (1987) The relation between major histocompatibility complex (MHC) restriction and the capacity of Ia to bind immunogenic peptides. Science **235:** 1353–1358.

Cambier J.C., Justement L.B., Newell K., Chen Z.Z., Harris L.K., Sandoval V.M., Klemsz M.J., and Ransom J.T. (1987) Transmembrane signals and intracellular "second messengers" in the regulation of quiescent B-lymphocyte activation. Immunol. Rev. **95:** 37–58.

Cease K.B., Berkower I., York-Jolly J., and Berzofsky J.A. (1986) T cell clones specific for an amphipathic alpha helical region of sperm whale myoglobin show differing fine specificities for synthetic peptides: A multi-view/single structure interpretation of immunodominance. J. Exp. Med. **164:** 1779–1784.

Collawn J.F., Bhayani H., and Patterson Y. (1989) An analysis of the physical properties of peptides that influence pigeon Cytochrome C specific T lymphocyte responses. Mol. Immunol. **26:** 1069–1079.

Corradin G., and Harbury H.A. (1970) Cleavage of cytochrome C with cyanogen bromide. Biochim. Biophys. Acta **221:** 489–496.

Crepaldi T., Crump A., Newmann M., Ferrone S., and Antczak D.F. (1986) Equine T lymphocytes express MHC Class II antigens. J. Immunogenet. **13:** 349–360.

Dottavio-Martin D., and Ravel M. (1978) Radiolabelling of proteins by reductive alkylation with $^{14}C$-formaldehyde and sodium cyanoborohydride. Anal. Biochem. **87:** 562–565.

Faulman E., Cuchens M.A., Lobb C.J., Miller N.W., and Clem L.W. (1983) An effective culture system for studying *in vitro*

mitogenic responses of channel catfish lymphocytes. Trans. Amer. Fish Soc. **112:** 673–679.

Flajnik M.F., Ferrone S., Cohen N., and Du Pasquier L. (1990) Evolution of the MHC: Antigenicity and unusual tissue distribution of *Xenopus* (Frog) Class II molecules. Mol. Immunol. **27:** 451–462.

Fu S.M., Chorazzi N., Wang C.W., Montazeri G., Kunkel H.G., Ko H.S., and Gottieb A.B. (1978) Ia bearing T lymphocytes in man. Their identification and role in the generation of allogeneic helper activity. J. Exp. Med. **148:** 1423–1428.

Gosselin E.J., Tony H.P., and Parker D.C. (1988) Characterization of antigen processing and presentation by resting B lymphocytes. J. Immunol. **140:** 1408–1413.

Heber-Katz E., Hansburg D., and Schwartz R. H. (1983) The Ia molecule of the antigen presenting cell plays a critical role in the immune response gene regulation of T cell activation. J. Mol. Cell. Immunol. **1:** 3–14.

Kojima M., Cease K.B., Buckenmeyer G.K., and Berzofsky J.A. (1988) Limiting dilution comparison of repertoires of high and low responder MHC-restricted T cells. J. Exp. Med. **167:** 1100–1113.

Kroemer G., Bernot A., Behar G., Chausse A.M., Gastinel L.N., Guillemot F., Park I., Thoraval P., and Auffray C. (1990) Molecular genetics of the chicken MHC: Current status and evolutionary aspects. Immunol. Rev. **113:** 119–145.

Lechler R.I., Lombardi G., Batchelor J.R., Reinsmoen N., and Bach F.H. (1990) The molecular basis of alloreactivity. Immunol. Today **11:** 83–88.

Miller N.W., Bly J.E., van Ginkel F., Ellsaesser C.F., and Clem L.W. (1987) Phylogeny of lymphocyte heterogeneity: Separation of functionally distinct subpopulation of channel catfish lymphocytes with monoclonal antibodies Dev. Comp. Immunol. **11:** 739–747.

Miller N.W., and Clem L.W. (1984) Microsystem for *in vitro* primary and secondary immunization of channel catfish (*Ictalurus punctatus*) leucocytes with hapten-carrier conjugates. J. Immunol. Methods **72:** 367–379.

Miller N.W., and Clem L.W. (1988) A culture system for mitogen-induced proliferation of channel catfish (*Ictalurus punctatus*) peripheral blood lymphocytes. J. Tissue Cult. Methods **11:** 69–73.

Miller N.W., Deuter A., and Clem L.W. (1986) Phylogeny of lymphocyte heterogeneity: Cellular requirement for the mixed leucocyte reaction in channel catfish. Immunology **59:** 123–128.

Pernis B., Silverstein S.C., and Vogel H.J., eds. (1988) Processing and presentation of antigens (New York: Academic Press).

Pierce S.K., Morris J.F., Grusby M.J., Kaumaya P., Buskirk A.V., Srinivasan M., Crump B., and Smolenski L.A. (1988) Antigen-presenting functions of B lymphocytes. Immunol. Rev. **106:** 149–180.

Schook L.B., and Tew J.G., eds. (1988) Antigen presenting cells: Diversity, differentiation and regulation (New York: Alan R. Liss).

Sercarz E.E., and Berzofsky J.A., eds. (1987) Immunogenicity of protein antigens: Repertoire and regulation, Vols. I and II (Boca Raton, FL: CRC Press).

Shastri N., Oki A., Miller A., and Sercarz E.E. (1985) Distinct recognition phenotypes exist for T cell clones specific for small peptide regions of proteins. Implications for the mechanisms underlying major histocompatibility complex-restricted antigen recognition and clonal deletion models of immune response gene defects. J. Exp. Med. **16:** 332–345.

Sizemore R.C., Miller N.W., Cuchens M.A., Lobb C.J., and Clem L.W. (1984) Phylogeny of lymphocyte heterogeneity: The cellular requirement for *in vitro* mitogenic responses of channel catfish leucocytes. J. Immunol. **133:** 2920–2924.

Smith-Gill S., and Sercarz E.E., eds. (1989) The immune responses to structurally defined proteins: The lysozyme model (New York: Adenine Press).

Vallejo A.N., Ellsaesser C.F., Miller N.W., and Clem L.W. (1990a) Spontaneous development of functionally active long term monocyte-like cell lines from channel catfish. In Vitro Cell. Dev. Biol. In press.

Vallejo A.N., Miller N.W., Jorgensen T., and Clem L.W. (1990b) Phylogeny of immune recognition: Antigen processing/presentation in channel catfish immune responses to hemocyanins. Cell. Immunol. **130:** 364–377.

